# ATX1-Generated H3K4me3 Is Required for Efficient Elongation of Transcription, Not Initiation, at ATX1-Regulated Genes

**DOI:** 10.1371/journal.pgen.1003111

**Published:** 2012-12-20

**Authors:** Yong Ding, Ivan Ndamukong, Zaoshi Xu, Hanna Lapko, Michael Fromm, Zoya Avramova

**Affiliations:** 1 School of Life Sciences, University of Science and Technology of China, Hefei, Anhui, China; 2 School of Biological Sciences, University of Nebraska at Lincoln, Lincoln, Nebraska, United States of America; 3 Department of Microbiology and Immunology, Brody School of Medicine, East Carolina University, Greenville, North Carolina, United States of America; 4 University of Nebraska Center for Biotechnology, Lincoln, Nebraska, United States of America; 5 Center for Plant Science Innovation, Lincoln, Nebraska, United States of America; The University of North Carolina at Chapel Hill, United States of America

## Abstract

Tri-methylated H3 lysine 4 (H3K4me3) is associated with transcriptionally active genes, but its function in the transcription process is still unclear. Point mutations in the catalytic domain of ATX1 (ARABIDOPSIS TRITHORAX1), a H3K4 methyltransferase, and RNAi knockdowns of subunits of the AtCOMPASS–like (Arabidopsis Complex Proteins Associated with Set) were used to address this question. We demonstrate that both ATX1 and AtCOMPASS–like are required for high level accumulation of TBP (TATA-binding protein) and Pol II at promoters and that this requirement is independent of the catalytic histone modifying activity. However, the catalytic function is critically required for transcription as H3K4me3 levels determine the efficiency of transcription elongation. The roles of H3K4me3, ATX1, and AtCOMPASS–like may be of a general relevance for transcription of Trithorax-activated eukaryotic genes.

## Author Summary

We provide a definitive answer to the question regarding the role of histone H3 lysine 4 tri-methylation marks in the transcription of two ATX1-regulated genes. Despite the proven correlation between the gene transcriptional activity and the level of H3K4me3 modification on the nucleosomes, whether H3K4me3 contributes to, or simply “registers,” active transcription has remained unclear. Another broader-relevance question is whether histone-modifying proteins are required for recruitment of the general transcription machinery, thus playing roles beyond their catalytic activity. Using a combination of gene deletion and specific point mutation analyses, we untangle overlapping effects and reveal that H3K4me3 is not required for TBP/Pol II recruitment to promoters but is critical as an activating mark for transcription elongation. The existing hitherto ambiguity about the role of H3K4me3 as an activating mark has been largely due to the unknown duality of the ATX1/AtCOMPASS functions: facilitating PIC assembly and producing H3K4me3 as an activating mark for transcription elongation.

## Introduction

The H3K4me3 mark is generally associated with transcriptionally active genes [1]–[3]. Its genome-wide distribution in yeast, animal, and plant genomes displays remarkably conserved, predominantly gene-associated, patterns with a strong bias towards the 5′-ends of transcribed genes [4]–[7]. Despite the demonstrated ability of chromatin remodeling/modifying and mRNA processing proteins to bind the H3K4me3 modification, the actual contribution of H3K4me3 to transcription is still unclear [8], [9].

The eukaryotic histone methyltransferases responsible for the H3K4me3 mark have diverged both evolutionarily and functionally into two families [10]. The TRITHORAX (TRX) family including Drosophila trithorax (Trx), mammalian MLL1-4, and Arabidopsis ATX 1-2 segregate into a phylogenetic subgroup that is distinct from the SET family containing yeast Set1 and its orthologs in other species [11]. SET family members operate more globally across the genome while the TRX family members are more gene-specific. In yeast, Set1is the sole methyltransferase establishing the genome-wide mono-, di-, and tri-methyl H3K4 marks, while MLL1 tri-methylates less than 5% of human genes [12], [13]. Like MLL1, ATX1 tri-methylates H3K4 at specific genes, but is not responsible for overall nucleosome modifications in Arabidopsis [14].

The mechanism of ATX1-dependent gene regulation in Arabidopsis involves features that are both similar and different from yeast Set1 and mammalian MLL models. A distinguishing feature of ATX1-dependent gene regulation is that ATX1 has dual roles upstream and downstream of the transcription start sites (TSS) of regulated genes [15]. At promoters ATX1 is found in a complex with TBP and Pol II affecting the formation/stability of the transcription preinitiation complex (PIC). The second role is within the transcribed region where ATX1 establishes a peak of H3K4me3 modified nucleosomes about 300 bp downstream of the TSS. ATX1's recruitment and ability to tri-methylate nucleosomes in this region requires the activated form of Pol II (phosphorylated at its carboxyl terminal domain (CTD) repeat at serine 5 (Ser5P) [15]. ATX1 binds directly to Ser5P Pol II, an interaction different from the Paf (Polymerase associated factor)-mediated binding of Set1/COMPASS to Ser5P Pol II in yeast [16].

Both SET and TRX family proteins (including the human, Drosophila, and Arabidopsis counterparts) operate within specific complexes, called COMPASS or COMPASS-LIKE, respectively. Both types of complexes share three conserved subunits, WDR5- ASH2L- RbBp5 that are critical for methyltransferase activity of the respective SET or TRX catalytic subunit [17]–[20]. The structural organization and the mechanism by which these three subunits stimulate the enzyme activity and H3K4me3 accumulation have been actively pursued and a significant amount of data for the biochemical and molecular mechanisms is available [21]–[23].

Although it has been well established that knockdown or deletion of a COMPASS or COMPASS-like subunit results in reduced mRNA and H3K4me3 levels of specific genes [17]–[20], how the specific stages of transcription are affected by this deficiency is less understood. Recently, the Drosophila Set1 (dSet1) was shown to be required for efficient release of Pol II into transcription elongation from the *heat shock 70* (*hsp70*) gene [24]. However, the roles of the TRX (MLL or ATX1) type COMPASS-like complexes in the transcription process have not been fully elucidated. Due to the structural and functional differences between the SET and the TRX family members [11], as well as differences in the protein composition and the interaction between the respective subunits of the COMPASS or COMPASS-like complexes [9], it is expected that the complexes supporting the activity of Set1 (including the human and Drosophila homologs) and those for TRX (MLL/ATX1) have functionally diversified as well.

Here, we study the roles of the Arabidopsis ATX1/COMPASS-like during the specific stages of transcription of two ATX1-regulated genes, W*RKY70* (*AT3G56400*, encoding a member of the WRKY family of transcription factors) and *LTP7* (*AT2G15050*, encoding a lipid transfer protein from an antimicrobial peptide family) [25]. ATX1 establishes the H3K4me3 marks at the 5′-end nucleosomes of these genes and is required for their optimal expression in leaves under regular homeostatic conditions [14]. Earlier, it has been reported that ATX1/AtCOMPASS–like affects transcript levels from the developmentally regulated *FLC* gene [20], [26]. However, how specific stages of transcription are affected has not been elucidated.

To distinguish the discrete transcription stages dependent on H3K4me3 levels from effects caused by the structural disruption of ATX1/AtCOMPASS–like, we used a combination of RNAi-mediated knockdowns of AtCOMPASS–like subunits and specific point mutations to inactivate the catalytic domain of ATX1. We demonstrate that ATX1, AtCOMPASS–like, and H3K4me3 have distinct effects on PIC formation and the transition to transcription elongation. ATX1 and AtCOMPASS–like are required for efficient PIC formation. In contrast to the MLL1-regulated gene model [27], the ATX1-generated H3K4me3 mark is not required for TBP recruitment during transcription initiation, but is critical for activating transcription elongation.

## Results

### AtCOMPASS–like regulates H3K4me3 and transcript levels of two ATX1-dependent genes

**Figure 1:**
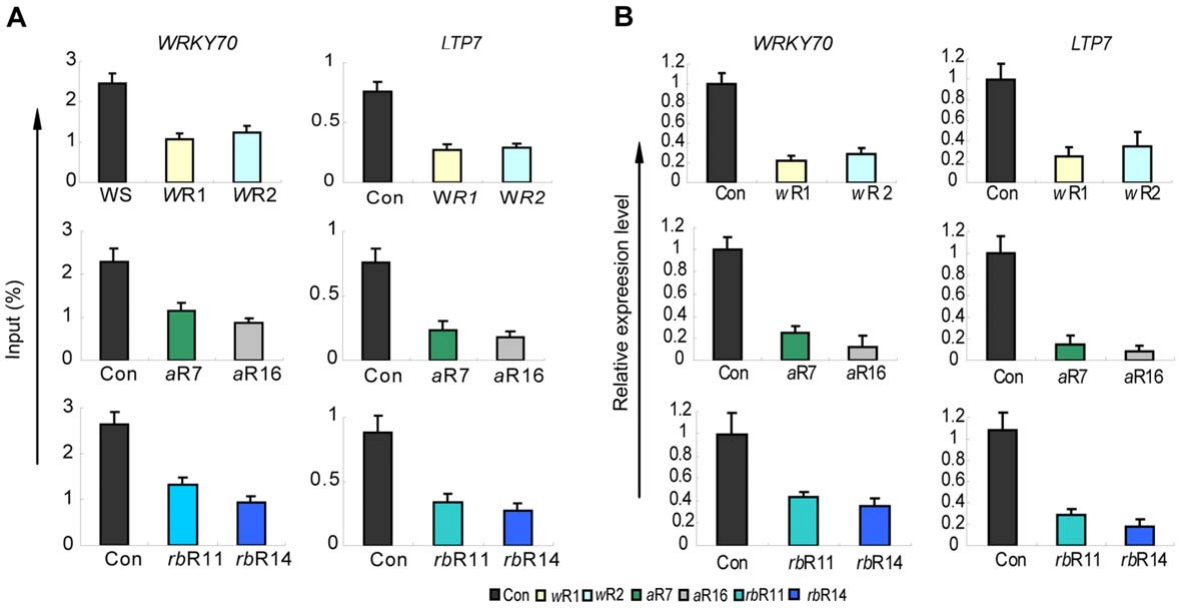
H3K4me3 and mRNA levels of two ATX1-regulated genes in the AtWRD5, or AtASH2, or At5-deficient lines. A) Relative H3K4me3 levels at *WRKY70* and *LTP7* in control wild type plants transformed with the empty vector (Con), in *AtWRD5-RNAi* (*w*R1, *w*R2) lines (top panels), *AtASH2-RNAi* (*a*R7, *a*R16) lines (middle panels), and *AtRbBp5a-RNAi* (*rb*R11, *rb*R14) lines (bottom panels) as measured by ChIP-PCR with HK4me3-specific antibodies. For characterization of the RNAi lines see SF 1A, B). Specific primers used for each gene are at the 5′-end, at the peak of H3K4me3 accumulation (Region 2, as indicated in Figure 2A). Background levels for immunoprecipitated samples with IgG as a control in all genetic backgrounds were <0.001 of the input levels (not shown). *ACT 7* is used as an internal control; B) Relative transcript levels produced by the genes in the RNAi knockdown lines described above. Each experiment was repeated at least three times. Each bar is standard errors of the mean (±SEMs, n = 3).

Two Arabidopsis proteins, AtWDR5a and AtWDR5b, are related to WDR5 but only AtWDR5a can form a complex with the other AtCOMPASS–like subunits [20]. We refer to AtWDR5a as AtWDR5 from here on. To analyze the function of the three core AtCOMPASS–like subunits, we generated plants expressing *AtWDR5*-*RNAi*, *AtASH2*-*RNAi* or *AtRbBp5*-*RNAi* constructs. Knockdown lines produced less transcripts from the respective subunit genes, confirming efficient knockdown of their target mRNAs (Figure S1A). The *AtWDR5*-*RNAi*, *AtASH2*-*RNAi* and *AtRbBp5*-*RNAi* knockdown lines displayed early flowering phenotypes similar to the *atx1* phenotype, supporting their function in a shared complex (Figure S1B; [20]). Lower expression of any of the AtCOMPASS–like subunits in the respective *AtWDR5*, *AtASH2*, or *AtRbBp5 RNAi* knockdown lines resulted in significantly reduced H3K4me3 levels at the 5′-ends of the W*RKY70* and *LTP7* genes known to be direct targets of ATX1 (Figure 1A). The W*RKY70* and *LTP7* genes also produced significantly reduced transcript levels in these knockdown lines (Figure 1B). We conclude that each of the three core AtCOMPASS–like subunits (AtWDR5, AtASH2, and AtRbBp5) must be present for the wild type H3K4me3 and transcript levels from the ATX1-regulated W*RKY70* and *LTP7* genes.

### Recruitment and distribution patterns of AtCOMPASS–like at ATX1-regulated genes

**Figure 2:**
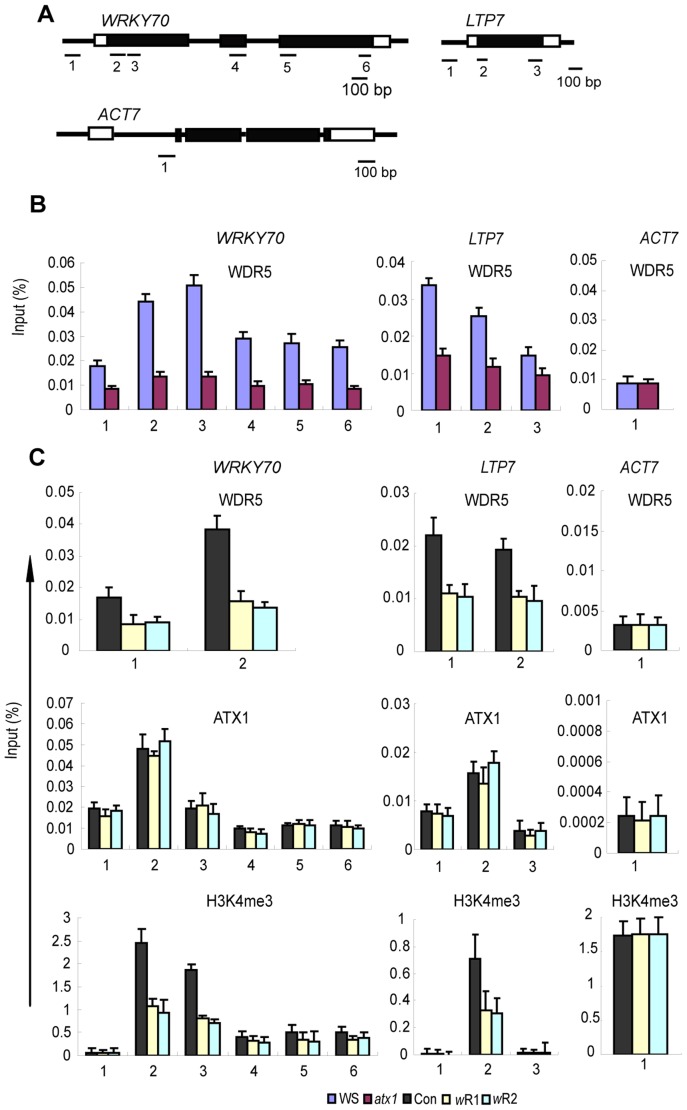
Distribution profiles of AtWDR5, ATX1, and H3K4me3 at the ATX1-regulated genes in different genotypes. A) Schematic diagram of the ATX1-regulated *WRKY70* and *LTP7* genes as well as the ATX1-independent *ACT7* gene. The 5′ or 3′ untranslated regions are shown as open boxes, the exons as black boxes, and the introns as thin black lines. Numbers below show the locations of the regions analyzed by ChIP-PCR; B) Distribution and amounts of AtWDR5 determined by ChIP-PCR with antibodies against WDR5 in WS (blue columns) and *atx1* (red columns) backgrounds; C) ChIP-PCR of WDR5, ATX1, and H3K4me3 in WS and AtWDR5 knockdown lines (*wR1,wR2*). Numbers on the X-axis show amplified regions as indicated in A. ChIP-PCR values with IgG in all genetic backgrounds were <0.001 of the input levels (not shown). Each experiment was repeated at least three times. Each bar is standard errors of the mean (±SEMs, n = 3).

The presence and the distribution patterns of AtWDR5 at the two ATX1-regulated genes were determined by ChIP analysis with antiWDR5 antibodies. AtWDR5 was found at the promoters and at the transcription start sites (TSS) regions of W*RKY70* and *LTP7* (Figure 2A, 2B). AtWDR5 accumulation peaked at the 5′-ends, then gradually tapered off downstream, in a profile similar to that of ATX1 (Figure 2C middle row) and H3K4me3 (Figure 2C, bottom row). The overlapping distribution patterns of ATX1 and AtWDR5 are consistent with a function in a shared complex. In addition, ATX1 and AtWDR5 interact directly in the yeast two-hybrid (Y2-H) binding system (Figure S2C; also shown in [20]) and a TAP-tagged AtWDR5 fusion protein used as bait in a pull-down assay successfully recovered ATX1 from total cellular protein extracts (for more details see Methods and Figure S2A).

The ATX1-AtWDR5 interaction was mapped further by Y2-H analysis and in pull down assays (Figure S2C, S2D; see Text S1). Detailed analysis of retained fragments (Figure S2A) by mass spectroscopy (Figure S3A–S3C) confirmed the ATX1 domain necessary and sufficient to bind to AtWDR5 was located immediately upstream of the SET domain (Figure S2B–S2D) and was similar (Figure S3D) to the Win (WDR5-interacting) peptide of MLL1 [21], [22]. This result is important as it indicates that ATX1 in Arabidopsis interacts with AtWDR5 through a conserved domain similar to the MLL1-WDR5 interaction in mammalian cells [28], [29].

Next, we determined whether ATX1 recruits AtCOMPASS–like (via AtWDR5) to the target genes or whether the presence of AtWDR5 was needed for recruiting ATX1. The AtWDR5 levels at the W*RKY70* and *LTP7* genes in *atx1* mutant and wild type backgrounds, determined by ChIP-PCR with antiWDR5 antibodies, were strongly diminished in *atx1* relative to the wild type background (Figure 2B). The results indicated ATX1 was required for wild type level occupancy of AtWDR5 at these genes. Thereby, AtWDR5 occupancy is dependent on ATX1 presence at the ATX1-regulated genes and, most likely, ATX1 helps recruit AtCOMPASS–like via binding to AtWDR5.

To determine whether ATX1 presence at the ATX1-regulated genes can occur independently of AtCOMPASS–like, we analyzed ATX1 levels by ChIP-PCR with antiATX1 antibodies in plants depleted for AtWDR5. First, we confirmed that in the *AtWDR5*-*RNAi* knockdown lines (Figure S1A) the amounts of AtWDR5 protein at the target gene loci was strongly reduced (Figure 2C, top row). We found that the amounts of ATX1 bound at the target genes in these *AtWDR5*-depleted lines were similar to their levels in the wild type (Figure 2C, middle row). We conclude that recruitment of AtWDR5 requires ATX1 but the converse is not true: ATX1 occupancy does not depend on AtWDR5.

Interestingly, the amount of ATX1 at the 5′-end regions of the target genes was slightly lower in *AtASH2-RNAi* lines (Figure S4), possibly suggesting an indirect effect of AtASH2, as AtASH2 does not directly interact with ATX1 (Figure S2C). Likewise, MLL1 does not bind Ash2L directly, but Ash2L is required for maintaining the integrity of the complex at the *HOX* loci [30].

### AtCOMPASS–like regulates transcription rates

**Figure 3:**
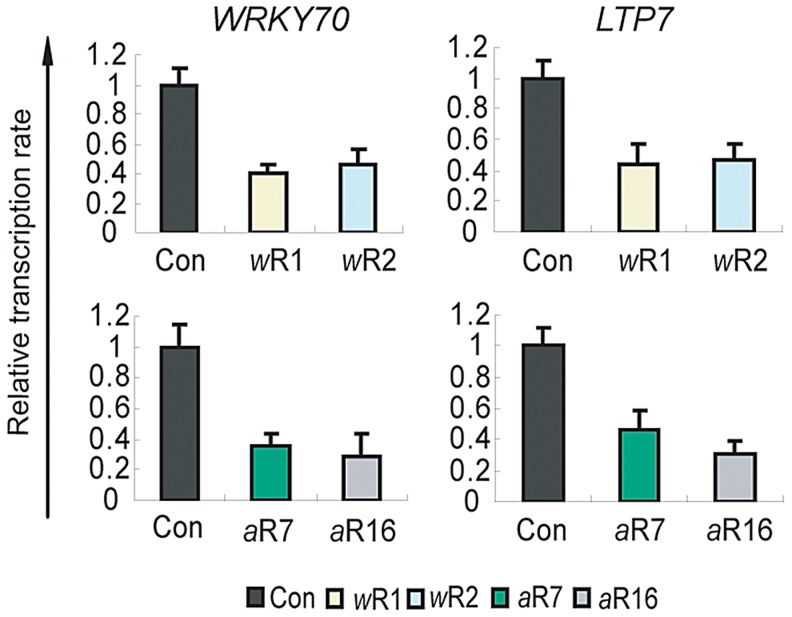
Role of AtCOMPASS–like in the transcription rates of the ATX1-regulated genes. Transcription rates of the ATX1-regulated genes determined by nuclear run on assays in empty vector (Con), RNAi knockdown plants deficient for AtWDR5 (*w*R1 and *w*R2) or AtAsh2 (*a*2R7 and *a*2R16). Each experiment was repeated at least three times. Each bar is standard errors of the mean (±SEMs, n = 3).

To determine whether the reduced transcript levels at the two target genes in the RNAi knockdown lines (Figure 1B) were due to defects in transcription or resulted from post-transcriptional events, we measured their rates of transcription. Nuclear run-on assays indicated that in the *AtWDR5*-*RNAi* and *AtASH2*-*RNAi* knockdown lines the W*RKY70* and *LTP7* genes were transcribed at much lower rates than in wild type (Figure 3). The reduction in the transcription rates is large enough to indicate that a reduced rate of transcription is the primary defect causing lower W*RKY70* and *LTP7* transcript levels in the *AtWDR5*-*RNAi* and *AtASH2*-*RNAi* knockdown lines.

### Roles of AtCOMPASS–like at promoters during initiation of transcription

**Figure 4:**
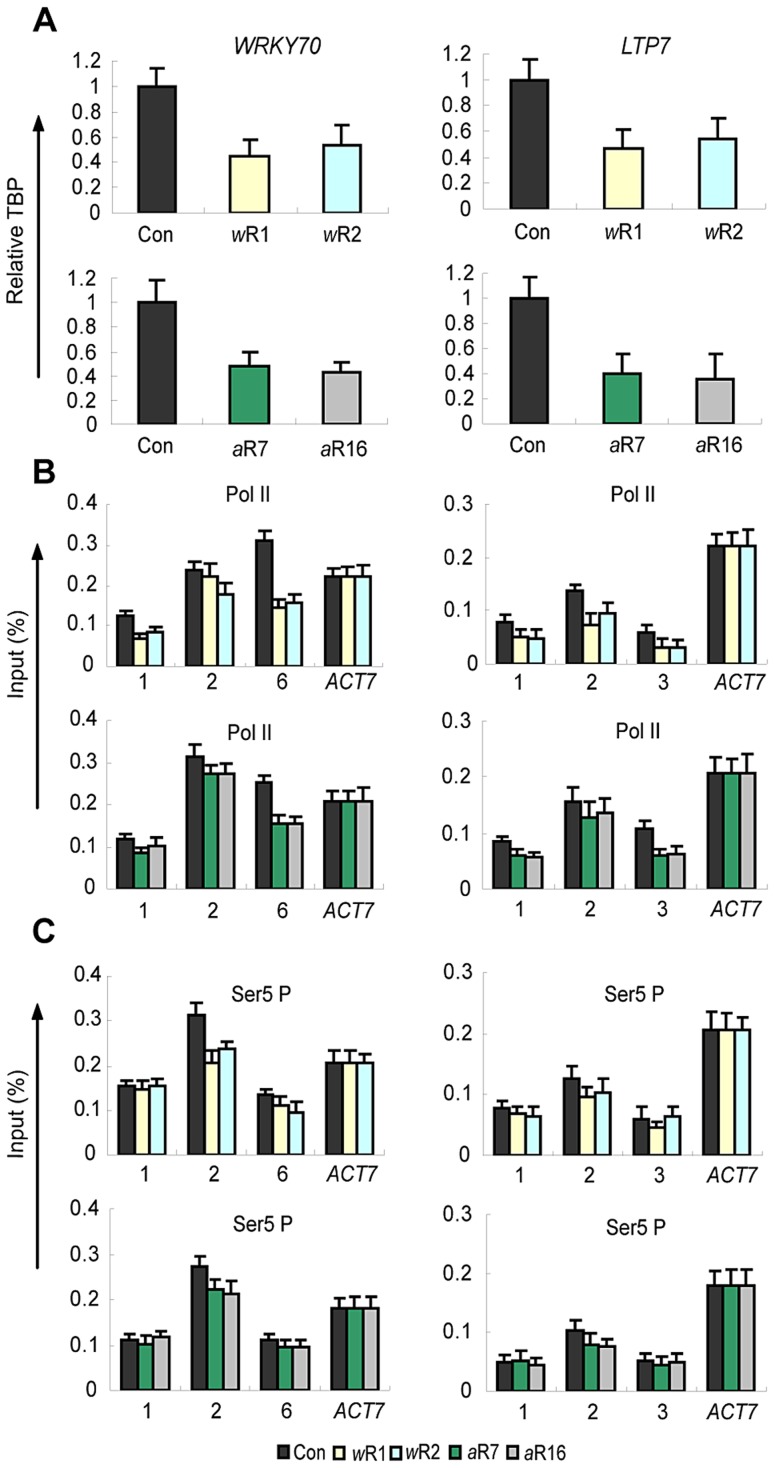
Distribution patterns and levels of TBP, of total RNA Pol II, and of Ser5P Pol II. A) Relative TBP levels at the promoters of the *WRKY70* and *LTP7* genes in empty vector control (Con) plants, in *AtWRD5-RNAi* (*w*R1, *w*R2) lines (top panel) and the *AtASH2-RNAi* (*a2*R7, *a2*R16) lines (bottom panel). *ACT 7* is used as an internal control; B–C) total Pol II and Ser5P distribution profiles along the genes in control (Con) and RNAi knockdown genotypes as specified above. Amplified regions are numbered as indicated in Figure 2A. ChIP-PCR values with IgG in all genetic backgrounds were <0.001 of the input levels (not shown). Each experiment was repeated at least three times. Each bar is standard errors of the mean (±SEMs, n = 3).

To gain insights into the role of AtCOMPASS–like in specific stages of transcription, we examined a possible role at the promoters by measuring TBP accumulation in the RNAi knockdown lines. Decreased *AtWDR5* or *AtASH2* mRNA levels correlated with ∼50% decrease in TBP levels (Figure 4A) suggesting an involvement of AtCOMPASS–like in TBP/PIC assembly.

Reduced TBP levels were likely to be associated with reduced Pol II recruitment. Therefore, we examined the occupancy of total Pol II at the analyzed genes. Total Pol II levels in *AtWDR5-RNAi* or *AtASH2-RNAi* knockdown lines were measured by ChIP with antibodies that do not discriminate between the non-phosphorylated and phosphorylated forms of Pol II (Figure 4B). Total Pol II accumulation at the 5′-ends of the three genes ranged from 64%–81% of wild type levels (Table 1).

**Table 1:** Distribution of total Pol II or its phosphorylated forms in RNAi COMPASS subunit-knockdown lines.

Gene	Pol II^1^	% 5′-end	% 3′-end	*p*-value (5′ vs 3′)
***WRKY70***	total	85±7	55±8	0.01
	Ser5P	75±7	-	
	Ser2P	-	39±2	<0.01
***LTP7***	total	64±7	48±4	0.05
	Ser5P	77±3	-	
	Ser2P	-	44±4	<0.01

1 The average of the ChIP-PCR values ± SD, n = 4, for w*dr5R1*, *wdr5R2*, *ash2R7*, and *ash2R16 RNAi* knockdown lines, as a percent of wild type. The 5′ and 3′-end regions correspond to regions 2 and 6 in Figure 2; for the shorter *LTP7* gene the 3′ end corresponds to region 3. *P*-values are for the significance of the differences between the 5′-end and 3′-ends.

Next, we measured the amount of Pol II phosphorylated at serine 5 of the CTD repeat (Ser5P Pol II) as this modification marks the transition of Pol II from the promoter (promoter clearance) to the sites of transcription initiation [31]–[33]. Ser5P Pol II levels near the TSSs of the genes in the *AtWDR5*-*RNAi* or *AtASH2-RNAi* lines were 75%–85% of wild type levels (Figure 4C, Table 1) indicating that Pol II accumulation at the TSSs was affected less strongly than the TBP/Pol II levels at the promoters. The strong reductions in the genes' transcript levels and transcription rates in the RNAi lines (Figure 4B), and the presence of relatively high total Pol II and/or Ser5P Pol II at the promoters and the 5′-ends of the genes (Figure 4B, 4C) suggested that disruption of AtCOMPASS–like also affected transcription downstream of these stages of transcription.

### Role of AtCOMPASS–like in transcription elongation

The amounts of total Pol II towards the genes' 3′-ends were measured to determine if transcription elongation was impaired. The total Pol II levels at the 3′-ends of the *WRKY70* and *LTP7* genes were strongly decreased in the RNAi lines (48%–55% of wild type levels, Figure 4B; Table 1). The differences between lower Pol II amounts at the 3(-ends and higher amounts at the 5(-ends were significant: (*p*-value(0.05 for *WRKY70* and *LTP7*). Less Pol II at the genes' 3(-ends suggested impaired transcription elongation.

**Figure 5:**
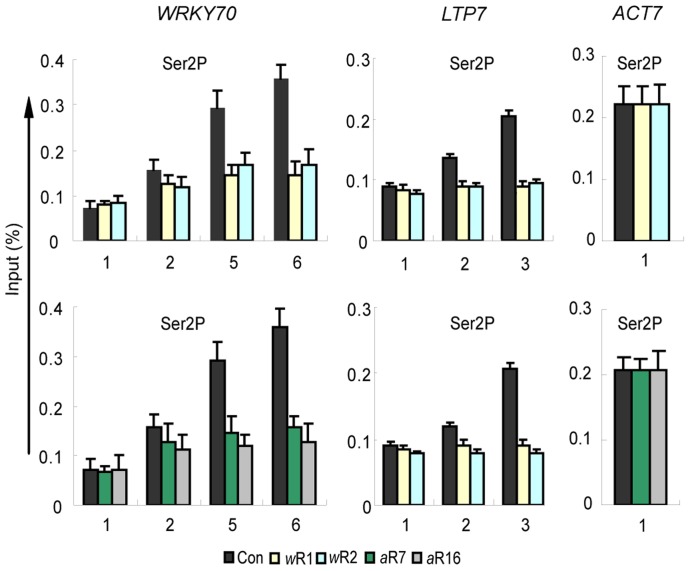
Ser2P Pol II distribution patterns along the genes in *AtWDR5* or *AtASH2-RNAi* knockdown lines. Distribution of Ser2P Pol II along the three genes in empty vector control (Con), *AtWRD5-RNAi* (*w*R1, *w*R2) and *AtASH2-RNAi* (*a*R7 and *a*R16) lines. ChIP-PCR values with IgG in all genetic backgrounds were <0.001 of the input levels (not shown). Each experiment was repeated at least three times. Each bar is standard errors of the mean (±SEMs, n = 3).

The distribution of Ser2P Pol II, which marks the transition of Pol II to the elongation phase [17], [32], [34], [35], was analyzed next. In the wild type background the Ser2P Pol II distribution increased towards the 3(-ends of the genes (Figure 5). In the *AtWDR5*-*RNAi* or *AtASH2*-*RNAi* knockdown lines, however, the Ser2P Pol II occupancy was considerably reduced (Figure 5). Importantly, the differences between the lower amounts of Pol II Ser2P at the 3′ ends and the higher amounts of Pol II Ser5P at the 5′ ends were significant (*p*-values<0.01, Table 1). We conclude that disruption of AtCOMPASS–like affected the transition from transcription initiation to transcription elongation.

Collectively, the results indicate that AtCOMPASS–like plays a role at the promoters (lower TBP and Pol II levels in *RNAi* lines), has a lesser effect on the Ser5P Pol II levels during the promoter clearance and transcription initiation, but is critically required for productive transcription elongation at the ATX-regulated genes.

### Catalytically inactive ATX1-set does not complement the *atx1* phenotype

The use of an *ATX1* T-DNA insertion mutant (*atx1*) or *RNAi* lines for depleting individual subunits of ATX1/AtCOMPASS–like decreases the amounts of both the intact complex and of H3K4me3, making it impossible to elucidate which of these alterations were affecting transcription. To distinguish the effects caused by changes in the structure of the ATX1/AtCOMPASS–like complex from effects caused by diminished H3K4me3 levels, we constructed an ATX1 mutant transgene, *ATX1-set*, containing point mutations expected to inactivate its catalytic methyltransferase domain while maintaining its structural integrity. This catalytically inactive *ATX1-set* gene contains 5 tyrosine to alanine mutations at positions that are evolutionarily conserved in ATX1 and MLL1 (see Methods). One of the conserved tyrosines (ATX1 Y1015) is known to be essential for the methyltransferase activity of the SET domain of human SET7/9 [36]. Expression of the HA-tagged ATX1-set protein in transgenic *atx1::ATX1-set* lines was verified by immunoblot analysis (Figure S5A). The failure of ATX1-set to rescue the early-flowering phenotype of *atx1* (Figure S5B) supports a deficiency in ATX1 function in the *atx1::ATX1-set* lines.

**Figure 6:**
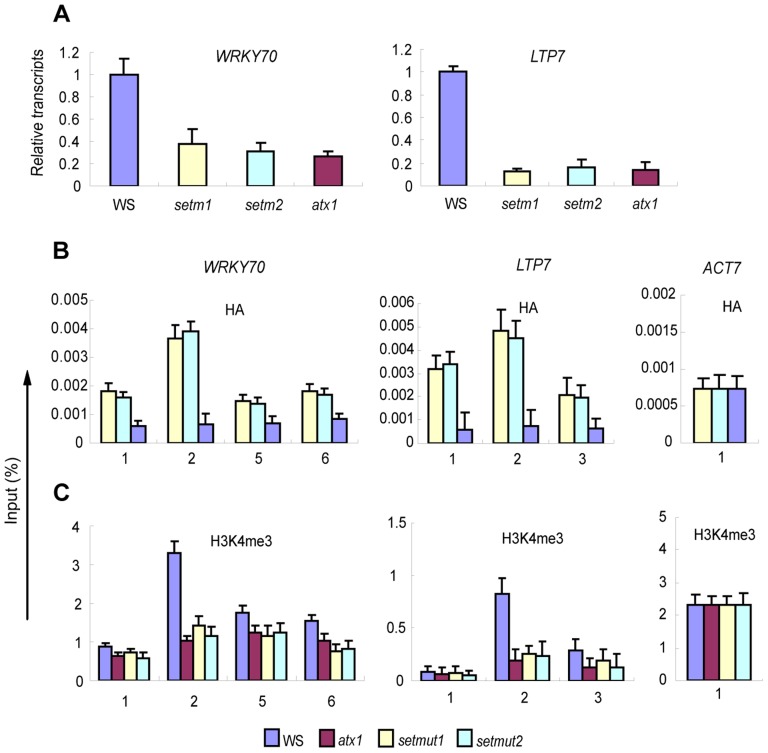
Transcriptional responses of *WRKY70* and *LTP7* genes and the amount of HA-tagged ATX1-set and H3K4me3 at these genes in different backgrounds. A) Transcript levels of *WRKY70* and *LTP7* genes in wild type (WS), two different *atx1:: ATX1-set* mutants (*setm1* and *setm2*, respectively; see SF 1), and *atx1* backgrounds; B) The amount of HA-tagged ATX1-set in the *atx1* background at the ATX1-regulated genes as determined by ChIP-PCR with antiHA antibody. Wild type (WS) was used as the non-specific control for the antiHA preciptiation as it lacks HA-tagged ATX1; C) Levels of H3K4me3 at the genes in wild type and the mutant backgrounds. Numbers on the x-coordinate represent regions along the gene sequences as indicated in Figure 2A, where specific primers were used in the ChIP-PCR assay. ChIP-PCR values with IgG in all genetic backgrounds were <0.001 of the input levels (not shown). Each experiment was repeated at least three times. Each bar is standard errors of the mean (±SEMs, n = 3).

Analyses of the transcriptional responses of ATX1-regulated genes in *atx1* plants expressing the *ATX1-set* transgene (*atx1::ATX1-set*) indicated the *WRKY 70* and *LTP7* transcripts were not restored to their wild type levels (Figure 6A). The reduced *WRKY 70* and *LTP7* transcription was due to the mutations in the catalytic domain and not the HA tag fusion protein structure as complementation of *atx1* with a HA-tagged version of wild type ATX1 restored the expression of these genes (Figure S5C). Complemented *atx1* plants also displayed the wild type flowering phenotype (Figure S5B) supporting the conclusion that the effects observed in the *atx1::ATX1-set* lines were caused by the deficient catalytic activity of ATX1-set.

One possible mechanism for the lower *WRKY 70* and *LTP7* transcription in the *atx1::ATX1-set* background could be the inability of ATX1-set to be recruited to its targets. ChIP assays with antiHA antibodies indicated the ATX1-set protein was located at the *WRKY 70* and *LTP7* genes (Figure 6B). However, despite the *ATX1-set* recruitment and accumulation at its targets, H3K4me3 levels were significantly lower than in wild type and comparable to the levels in *atx1* mutants (Figure 6C). We conclude that, although ATX1-set was present at the 5′-ends of its gene targets, the ATX1-set histone modifying activity was strongly decreased or absent. The next question, then, was whether ATX1-set could still recruit AtWDR5.

**Figure 7:**
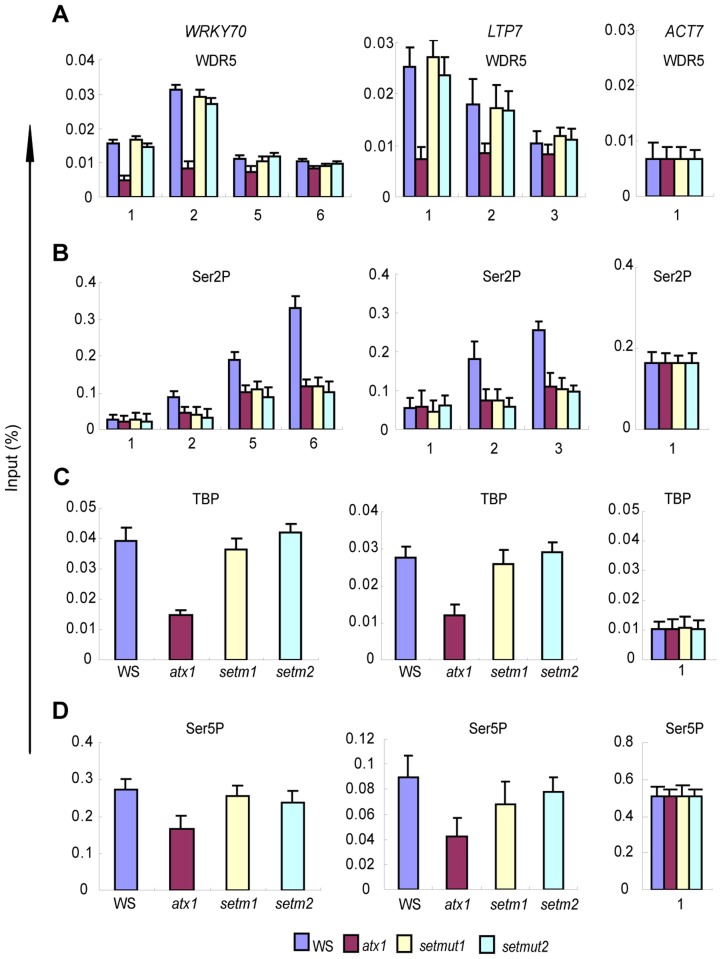
Occupancy of WDR5, TBP, Ser5P, and Ser2P Pol II in WS, atx1, or atx1::ATX1-set (setm1 and setm2) backgrounds. A) Occupancy by AtWDR5 at the promoters (region 1) and downstream regions; B) Distribution of Ser2P along the genes; C) Levels of TBP recruited to the promoters and D) of Ser5P Pol II at the promoters of the two genes in the different backgrounds. ChIP-PCR values with IgG in all genetic backgrounds were <0.001 of the input levels (not shown). Amplified regions are as indicated in Figure 2A). Each experiment was repeated at least three times. Each bar is standard errors of the mean (±SEMs, n = 3).

ChIP assays with antiWDR5 antibodies indicated that both the amounts and the distribution patterns of AtWDR5 were similar to those in wild type and significantly higher than in an *atx1* background (Figure 7A). The results indicated the ATX1-set protein maintained its structural integrity and ability to interact with the AtCOMPASS–like complex at its target genes. These results justify the use of the *atx1::ATX1-set* plants to assess how diminished H3K4me3 levels affected transcription without apparent changes in the other functions of the ATX1-set/AtCOMPASS–like complex.

### The role of H3K4me3 in transcription initiation and/or elongation

The diminished transcript and H3K4me3 levels at the *WRKY70* and *LTP7* genes in the *atx1::ATX1-set* background (Figure 6C) indicate this histone modification is a major factor responsible for the decreased transcript levels from these genes (Figure 6A). Next, we analyzed the effects of the diminished amounts of H3K4me3 in *atx1::ATX1-set* plants upon the levels of the elongating Ser2P Pol II. We found that the levels of Ser2P Pol II were low and comparable to the levels in the *atx1* background (Figure 7B), indicating overall transcription was diminished.

Whether the diminished H3K4me3 levels affected TBP recruitment to the promoters was analyzed in *atx1::ATX1-set* plants. TBP accumulated at the promoters to levels similar to wild type and much higher than in the *atx1* background (Figure 7C). Accumulation of Pol II in its initiation-activated form (Ser5P) at the *WRKY70* and *LTP7* promoters was nearly at wild type levels in *atx1::ATX1-set* and again at higher levels than in *atx1* (Figure 7D). The most important consequences of this result are that the absence of ATX1-generated H3K4me3 marks did not markedly interfere with the assembly of the basal transcriptional machinery and, that the primary defect in transcription was in the attenuated levels of Pol II Ser2P levels at the genes 3′ ends (Figure 7B).

Summarily, despite ‘normal’ recruitment of TBP, ATX1 and AtCOMPASS–like to the 5′-ends of the genes, the rates of transcription elongation were diminished when H3K4me3 levels were low, providing compelling evidence that H3K4me3 is an activating mark for elongation at ATX1/AtCOMPASS–like regulated genes.

## Discussion

A large body of published work has demonstrated that expressed genes have higher levels of tri-methylated H3K4 residues on their nucleosomes than non-expressed genes [12], [14], [37]–[39]. Although deficiencies in H3K4me3 via knockdown of COMPASS subunits result in reduced levels of mRNA production [17]–[20], the mechanisms by which H3K4me3 affects transcription are still emerging [8], [9]. Revealing a causative link between H3K4me3 and transcription has been particularly challenging, as histone methyltransferases are multidomain proteins that function within large protein complexes. Consequently, interpretation of results based on knockdown mutations may be misleading as the effects could result from disruption of the multiple functions of the protein and complexes involved. For example, mutating ATX1 affects the assembly or stability of TBP/PIC as well as H3K4 methylation at downstream nucleosomes [15]. These observations underscore the need for specific mutations that affect only one function while maintaining the structural integrity of the protein of interest.

### AtCOMPASS–like has dual roles in transcription

In addition to reported similarities of AtCOMPASS–like [20] with the extensively studied COMPASS/COMPASS-like complexes in animals and yeast, we establish a role for AtCOMPASS–like in transcription that has not been reported for yeast, fly or mammalian complexes. We demonstrate that AtCOMPASS–like, as shown earlier for ATX1 [15], has dual roles in transcription initiation and H3K4 tri-methylation. Specifically, AtCOMPASS–like is recruited to promoters by ATX1 (Figure 2B) and plays a role in TBP/PIC assembly and/or stability. Reduced amounts of AtWDR5 or AtASH2 caused ∼50% decreases in TBP levels at the promoters (Figure 4A), attenuated transcription, and reduced Pol II levels on the transcribed genes (Table 1). These results link the AtCOMPASS–like complex with the basal transcriptional machinery. In these studies, as in all studies reported for other systems, we have used RNAi to deplete subunits of AtCOMPASS–like. As a consequence, it was not possible to separate the effects of structurally disrupting AtCOMPASS–like from the effects resulting from low H3K4me3 levels. Here, we successfully uncouple the ATX1/AtCOMPASS–like structural contributions from changes in H3K4me3 levels through analysis of *atx1::ATX1-set* mutant plants. The point mutations in this ATX1-set mutant protein greatly diminish (or eliminate) methyltransferase activity *in vivo* as ATX1 target genes had H3K4me3 levels that were identical to those in the *atx1* background. The apparent structural integrity of the ATX1-set mutant supported, *first*, by its ability to be correctly recruited to its target genes and *second*, by its ability to efficiently recruit AtWDR5 (Figure 7A) and to recruit/stabilize TBP/Pol II to promoters (Figure 7C, 7D) despite an apparent lack of catalytic methyltransferase activity *in vivo*, allowed us to clearly separate the dual roles of ATX1/AtCOMPASS–like in transcription.

### ATX1/AtCOMPASS–like regulate transcription initiation independently of the H3K4me3 activity

The ATX1-regulated *WRKY70* and *LTP* genes in wild type, *atx1*, and *atx1::ATX1-set* mutant backgrounds displayed clear differences in their transcriptional behavior. The strongly reduced *WRKY70* and *LTP* transcript production in the absence of ATX1 in *atx1* mutants was consistent with lower TBP and Pol II occupancy at the promoters [15]. In contrast, in the *atx1::ATX1-set* mutant, the TBP/Pol II (PIC) levels at the promoters were similar to the wild type (Figure 7C, 7D). This result demonstrates that PIC levels at promoters depend on the structural integrity of ATX1 but not on its H3K4 methyltransferase activity. The results from this study, together with the finding of ATX1 in a protein complex with TBP and ATX1's ability to bind directly to the non-phosphorylated form of Pol II [15], define a novel role for ATX1/AtCOMPASS–like as a transcriptional co-activator separate and largely independent of its histone modifying activity. This model differs from the binding of the TAF3 subunit of TFIID to H3K4me3 at MLL1-regulated genes [27]. Additionally, the Arabidopsis genome lacks a TAF3 subunit [40], making anchoring of TFIID to H3K4me3 nucleosome an unlikely mechanism for ATX1-regulated genes.

It is important to note also that, hitherto, a role of histone modifying proteins in PIC formation, that is independent of their histone modification activity, has been found only for yeast histone acetyltransferases [41]–[43]. Our results provide the first demonstration of a histone methyltransferase as an essential component of the general transcription machinery independent of its methyltransferase activity.

Furthermore, as knockdown of the AtWDR5 or AtASH2 subunits reduced TBP occupancy by ∼50% at the promoters, it was surprising that the Ser5P Pol II levels at the 5′-ends of the genes was only slightly decreased (Figure 4C; Table 1). As Ser5P Pol II is a biochemical marker for transcription initiation and early elongation [44]–[46], the results indicated that although required for normal TBP levels, ATX1/AtCOMPASS–like had a lesser effect on Pol II levels after promoter clearance. A possible reason is that a rate-limiting step in transcription on these templates is downstream of these early stages.

### H3K4me3 is required for efficient transcription elongation

As the ATX1-dependent TBP levels at the promoters were similar to wild type, the attenuated transcript production in *atx1::ATX1-set* mutant lines indicated H3K4me3 was required for efficient transcriptional processes taking place after PIC formation. Together, the results showing relatively high TBP and Pol II levels at the genes' 5′ ends (Figure 4A–4C), reduced rates of transcription (Figure 3), and reduced amounts of Pol II and its elongating Ser2P form at the genes' 3′ ends (Figure 5, Table 1), indicate that transcription elongation is diminished in H3K4me3 deficient genes.

**Figure 8:**
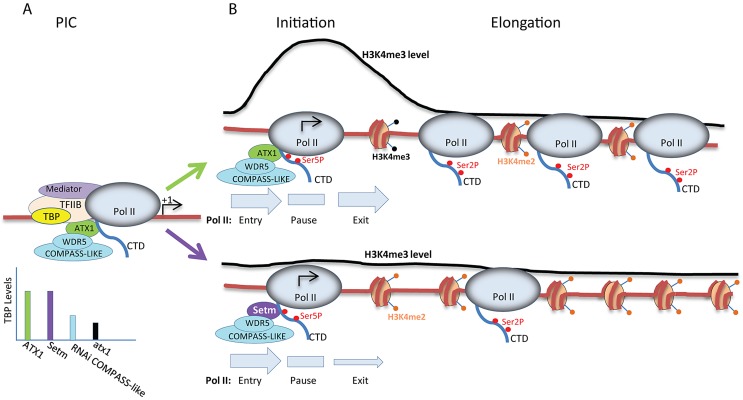
Model for the roles of ATX1/AtCOMPASS–like and H3K4me3 in transcription. A) The levels of PIC assembled at promoters depend on the integrity of ATX1/AtCOMPASS–like as ATX1 occurs in a complex with TBP (yellow) and interacts with the carboxyl terminal domain of Pol II (CTD, blue tail). The bar graph in A below the template shows the relative TBP levels that occur in wild type ATX1 (green), in the ATX1-setm mutant (Setm, in purple), in RNAi COMPASS-like knockdowns (blue), and in *atx1* (black) backgrounds; B) The transition to transcription initiation and promoter proximal pausing (gray pause rectangle) occurs with similar efficiencies (gray Pol II entry arrows of similar width) for both complexes that differ only in their ATX1 subunits: wild type ATX1 (top green ATX1) and ATX1-setm mutant (bottom purple Setm). Both wild type and the ATX1-setm ATX1/COMPASS-like complexes are recruited to this site via ATX1's affinity for the Ser5P form of Pol II. Wild type ATX1/COMPASS-like produce normal amounts of H3K4me3 levels, but H3K4me3 levels are diminished in the ATX1-setm mutant (the black line above the templates shows H3K4me3 profile). The levels of H3K4me3 affect the rate of Pol II exit from the promoter proximal pause region (size of gray Pol II exit arrows below the templates), with a higher exit rate for the wild type template. These different exit rates lead to more Pol II Ser2P complexes active in transcription elongation in ATX1 than in the ATX1-setm mutant.

Our results are consistent with a model (Figure 8) in which lower transcription and lower Ser2P Pol II amounts at the genes' 3′ ends are due to slow release of Pol II from a promoter proximal pause site into productive elongation. Diminished release would account for the accumulation of Ser5P Pol II at the genes' 5′ ends, relative to their 3′ ends, in agreement with the model suggested for the Drosophila *hsp70* gene when depleted of the dSet1 protein [24]. We suggest that H3K4me3 generated by either Set/COMPASS or TRX/COMPASS-like complexes plays similar roles in activating the transition to transcription elongation. However, for the Set/COMPASS it remains to be established whether it affects the basal transcriptional machinery similarly to the role found here for ATX1/AtCOMPASS–like.

Regulation of elongation is emerging as a critical mechanism for regulating transcription in developmentally regulated and heat-shock induced animal genes, where the limiting step is the release of paused/stalled Pol II into elongation [47]–[50]. It is important to emphasize a principle difference between animal genes regulated by paused/stalled Pol II and the ATX1-regulated genes reported here: these animal genes carry pre-accumulated Ser5P Pol II at their 5′-ends before entering active transcription and they require stimulation by additional factors to release them into productive elongation[33], [47], [49]. In contrast, the ATX1-regulated genes studied here are actively transcribed in non-stressed differentiated tissues and do not have paused/stalled Pol II at their 5′-ends. However, the genes experience accumulation of Ser5P Pol II downstream of TSS (as a form of pausing/stalling) when H3K4me3 levels are depleted. It is tempting to speculate that regulation of H3K4me3 levels is a more general mechanism controlling elongation not limited to inducible genes or genes with pre-stalled Pol II.

Summarily, we conclude that although the presence of ATX1/AtCOMPASS–like is required for assembly of the basal transcription machinery (transcription initiation) at the promoter, the H3K4me3 mark generated by ATX1/AtCOMPASS–like is not required for transcription initiation, but is an activating mark for transcription elongation. The mechanisms by which H3K4me3 affects transition to productive transcription elongation remain to be established. H3K4me3 may be responsible for the generation of a chromatin structure at the 5′-end to ensure optimal Pol II release into productive elongation and/or recruitment of pre-mRNA processing and elongation factors to the 5′ regions of genes [18], [51]–[53].

Lastly, our finding that the ATX1-Win domain is a functional counterpart of the Win domain in MLL1 (Figure S2, Figure S3) [21], [22], [54] suggests that ATX1 integrates into the AtCOMPASS–like exclusively through the Win-mediated binding to AtWDR5. This result underscores the relatedness of the ATX1/AtCOMPASS–like with the human MLL1/COMPASS-like [28]. Therefore, our results may have a broader relevance for the TRX-regulated genes in eukaryotes.

## Materials and Methods

Arabidopsis plants (WS ecotype) were grown for 14–21 d in potting soil in growth rooms at 22°C with a 12-h light photoperiod. Descriptions of all the cloning vectors and primers used in this study, as well as plasmid construction and generation of *AtWDR5a*-*RNAi*, *AtASH-RNAi*, and *AtRbBp5-RNAi* transgenic lines, are provided in ST 1. Genes used in this study have the following IDs: *ATX1* (*At2g31650*), *WDR5a (At3g49660), AtASH2 (At1g51450), AtRbBp5 (At3g21060), TBP(At3g13445)*, W*RKY70* (*AT3G56400*), *LTP7* (*AT2G15050*).

### Yeast two-hybrid assay

The AH190 strain was transformed with one of the following bait constructs: pGBKT-AtWDR5, pGBKT-AtASH2, or pGBKT-AtRbBp5, then transformed with one of the prey constructs: pGADT7-ATX1, pGADT7-ATX1N, pGADT7-ATX1C, pGADT7-ATX1DH, pGADT7-ATX1win, pGADT7-ATX1SET, pGADT7-AtAsh2, or pGADT7-AtRbBp5. Yeast were scored for protein interactions by their ability to grow on SD medium lacking Trp, Leu, His, and Ade.

### Protein pull down assays

For GST-bead pull down assays, GST beads were incubated with 2 µg of each GST fusion protein, washed, and then incubated with 3 µg of a His-fusion protein overnight at 4°C. Mock controls used extracts prepared from *E. coli* containing the His-Tag or GST vectors. The beads were washed five times (1×PBS buffer, pH 7.4, containing 140 mM NaCl, 1 mM PMSF and 0.1% TritonX-100), and the remaining proteins eluted from the washed beads in SDS-loading buffer, separated on a 12% PAGE/SDS gel, and analyzed by anti-GST (G018, Applied Biological Materials, Richmond, BC, Canada, lot: 5019) or anti-His antibody (05-949, Millipore, Lot:1487531).

### Nuclear run-on assays

Analyses were performed as described earlier [55].

### Chromatin immunoprecipitation assay

The ChIP assay was performed using a modified method [56]. Briefly, 3 g of leaves were fixed with 1% formaldehyde for 10 min and quenched in 0.125 M glycine. The treated leaves were ground in a mortar and pestle in liquid nitrogen, the resulting powder was solubilized in extraction buffer and filtered through miracloth. After sonication and centrifugation, the supernatant was pre-cleared with protein A magnetic beads (Invitrogen, Carlsbad, CA), and immunoprecipitated with one of the following antibodies recognizing: Pol II (ab817, Abcam, Cambridge, MA, Lot: 669648); the Ser2P form of Pol II CTD (ab5095, Abcam, Cambridge, MA, Lot: 703307); the Ser5P form of Pol II CTD (ab5131, Abcam, Cambridge, MA, Lot: 806890); trimethyl-H3K4 (ab8580, Abcam, Cambridge, MA, Lot: 598382); ATX1 (rabbit sera, GenScript, SC1031); AtWDR5 (ab75439, Abcam, Cambridge, MA, Lot:872536);); TBP (ab52887, Abcam, Lot:347607), or control IgG serum added for overnight incubation at 4°C. The antibody-protein complexes were isolated by binding to protein A or protein G beads. The washed beads were heated at 65°C for 8 h with proteinase K to reverse the formaldehyde cross-linking and digest proteins. The sample was then extracted with phenol/chloroform, the DNA precipitated in ethanol, and then re-suspended in water. Purified DNA was analyzed by real-time PCR with gene-specific primers. In all ChIP experiments DNA has been fragmented to 100–500 bp with the majority fragments having a length of 200–300 bp.

### ATX1-set mutant

The ATX1-setm mutant contains 5 tyrosine to alanine substitutions: Y927A, Y945A, Y954A, Y1013A, and Y1015A. The *ATX1-set* is a synthetic gene expressed from the MAS promoter and encoding the wild type ATX1 protein with a N-terminal HA fusion, except for the mutations noted above.

### Mass spectrometry and data analysis

A Q-TOF Ultima tandem mass spectrometer (Waters) with electrospray ionization was used to analyze the eluting peptides. The stained bands were excised and subjected to LC/MS as described [57]. Gel pieces were digested by trypsin (no. V5111, Promega, Madison, WI) and digested peptides were extracted in 5% formic acid/50% acetonitrile and separated using C18 reversed phase LC column (75 micron×15 cm, Pepmap 300, 5 micron particle size) (Dionex, Sunnyvale, CA). A Q-TOF Ultima tandem mass spectrometer (Waters) with electrospray ionization was used to analyze the eluting peptides. The system was user-controlled employing MassLynx software (v 4.1, Waters) in data-dependant acquisition mode with the following parameters: 0.9-sec survey scan (380–1900 Da) followed by up to three 1.4-sec MS/MS acquisitions (60–1900 Da). The instrument was operated at a mass resolution of 8000. The instrument was calibrated using the fragment ion masses of doubly protonated Glu-fibrinopeptide. The peak lists of MS/MS data were generated using Distiller (Matrix Science, London, UK) using charge state recognition and de-isotoping with the other default parameters for Q-TOF data. Data base searches of the acquired MS/MS spectra were performed using Mascot (Matrix Science, v1.9.0, London, UK). The NCBI non-redundant database (2010130-10386837 sequences 3543419944 residues) was used restricted to *Arabidopsis thaliana*. Search parameters used were: no restrictions on protein molecular weight or pI, enzymatic specificity was set to trypsin with up to 3 missed cleavage sites, carbamidomethylation of C was selected as a fixed modification. Mass accuracy settings were 0.15 daltons for peptide mass.

### Reverse transcription and real-time PCR

Total RNA isolation and reverse transcription with oligo(dT) (18418-012; Invitrogen, Carlsbad, CA) were performed as described previously [15], [58]. Transcript levels were measured with gene-specific primers by real-time PCR analysis with a cyclerIQ real-time PCR instrument (Bio-Rad, Hercules, CA) and SYBR Green mixture (Bio-Rad, Hercules, CA). The relative amount of specific gene transcripts was quantitated with the 2^−ΔΔ^Ct calculation according to the manufacturer's software (Bio-Rad, Hercules, CA), where ΔΔCt is the difference in the threshold cycles and the reference housekeeping gene; *ACT7* was used as an internal control for ChIP experiments and immunoprecipitated DNA was expressed as a percent of input DNA.

Primers used for the various cloning and analytical procedures are in Table S1.

## Supporting Information

Figure S1 A) Relative mRNA levels in the two transgenic *AtWDR5-RNAi* (*w*R1 and *w*R2), two transgenic *AtASH2-RNAi* (*a*2R7 and *a*2R16), and two transgenic *AtRbBP5-RNAi* (*rb*R11, *rb*R14) lines used in this study. Transgenic plants transformed with the empty vector (Con) were used as a control; B) Flowering phenotypes in the *RNAi*-lines, in *atx1*, and control backgrounds.
(PDF)

Figure S2 Interactions between ATX1, AtWDR5a, AtASH2, and AtRbPB5; Identification of the ATX1-Win AtWDR5-binding domain. A) Proteins from total cellular extracts retained by TAP-tagged-AtWDR5a in a pull-down experiment. Gel stained by Coomassie blue (left) and western blot assay with antiATX1 antibody (right). Arrows point to ATX1 (120 kD) and degradation products. Identities were established by MS (Figure S3); B) Schematic representation of ATX1 protein domains tested for their ability to bind AtWDR5a in a Y2-H system. Fragments corresponding to the ATX1 structural domains (PWWP, DAST, ePHD, Win, and SET) are indicated. Win (50 aa) represents a construct containing 50 amino acids upstream of the SET domain corresponding to the MLL1-Win peptide. C) Positive or negative interactions between ATX1 deletion fragments with AtWDR5a are indicated with (+) or (−), respectively. The experimental Y-2H data are shown on the right. The ATX1 proteins (shown in B) were fused to the activation domain (AD) and tested for their ability to bind to AtWDR5, AtASH2, or AtRbBP5, which were fused to the DNA binding domain (BD). Y-2H controls were performed with an AD only construct in combination with a BD containing construct (WDR5, ASH2, RbBP5, or BD alone) and the resulting growth or lack of growth of the yeast colonies is shown; D) In vitro pull-down assays of the domains tested in the Y2-H assays. Immobilized GST-tagged ATX1, or various ATX1-deletion fragments were tested for binding to soluble His-tagged AtWDR5 (top panel); Immobilized His-AtWDR5 was tested for binding to soluble GST-tagged ATX1 fragments (lower panel). Input and bound proteins were detected by anti-His tag or anti-GST antibody.
(PDF)

Figure S3 Identification of AtWDR5 interacting protein bands by MS. A) Coverage map of the ∼120 kD band reacting with the antiATX1 antibody (see arrow in Figure S2A). From MASCOT database search: Match to: gi|12659210 Score: 2058. Trithorax-like protein 1 [Arabidopsis thaliana]; Matched peptides shown in bold red. Score cut-off 35; B) Coverage map of the ∼72 kD band reacting with the antiATX1 antibody (see Figure 1A in text, arrowhead). From MASCOT database search: Match to: gi|12659210 Score: 3258. Trithorax-like protein 1 [Arabidopsis thaliana]; Matched peptides shown in bold red. Score cut-off 35; C) Coverage map of the ∼28 kD band reacting with the ATX1 antibody (see Figure 1A in text, arrow). From MASCOT database search: Match to: gi|12659210 Score: 863-Trithorax-like protein 1 [Arabidopsis thaliana]; Matched peptides shown in bold red. Score cut-off 35. Highlighted in yellow is the Win-homologous sequence; D) Alignment of the conserved Win seq upstream of the SET domain in MLL1 (Query) and ATX1 (Sbjct). The consensus ART sequence involved in the interaction with WDR5 is highlighted.
(PDF)

Figure S4 The amount of ATX1 at the promoter regions of three genes in AtASH2-deficient lines was determined by ChIP-PCR using antiATX1 antibody. The primers were located within the promoter regions (Region 1 in Figure 2A, main text).
(PDF)

Figure S5 Transgenic *atx1* mutant plants expressing the synthetic HA-tagged ATX1-wtSET domain protein or the HA-tagged ATX1 with Tyr/Ala substitutions in the SET domain (set). A) Western blot assay with antiHA antibody illustrating the expression of HA-ATX1-wtSET in two *atx1* transgenic lines (HA-ATX1 and HA-ATX2) and in two *atx1* lines expressing the HA-tagged ATX1-set mutant proteins (setm1 and setm2) transformed with the respective constructs. The levels of histone H3 expression detected by antiH3 antibodies are shown as loading controls; B) Flowering time phenotypes of plants from the transformed lines shown in (A). Transgenic *atx1* plants expressing the HA-ATX1-setm do not rescue the early flowering caused by the *atx1* mutation, while transformants expressing the wt SET restored wild type flowering; C) expression of the *WRKY70* and *LTP7* genes in *set1* and *set2* mutant lines in the *atx1* background and in the *atx1::HA-ATX1-wtSet* background. The higher transcript levels from the *WRKY70* and *LTP7* genes may result from higher ATX1 protein produced in the transgenic lines.
(PDF)

Table S1 Primers used for the various cloning and analytical procedures.
(PDF)

Text S1 Interaction between ATX1 and AtWDR5a; Identification of the ATX1-Win AtWDR5-binding domain. Interactions between ATX1 and AtASH2 or AtRbPB5.
(PDF)
