# Supplementary figures and images for: ATX1-Generated H3K4me3 Is Required for Efficient Elongation of Transcription, Not Initiation, at ATX1-Regulated Genes
Source: PLoS Genet. 2012 Dec 20;8(12):e1003111. doi: 10.1371/journal.pgen.1003111 (PMC3527332; doi:10.1371/journal.pgen.1003111)

Ding et al., Figure S1

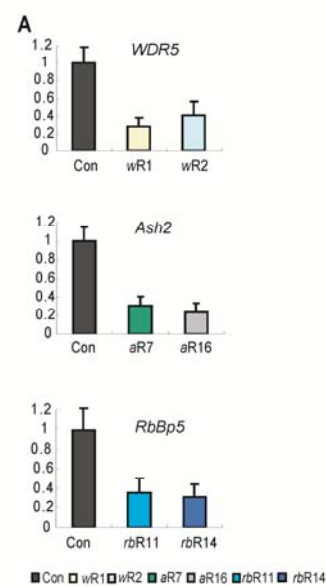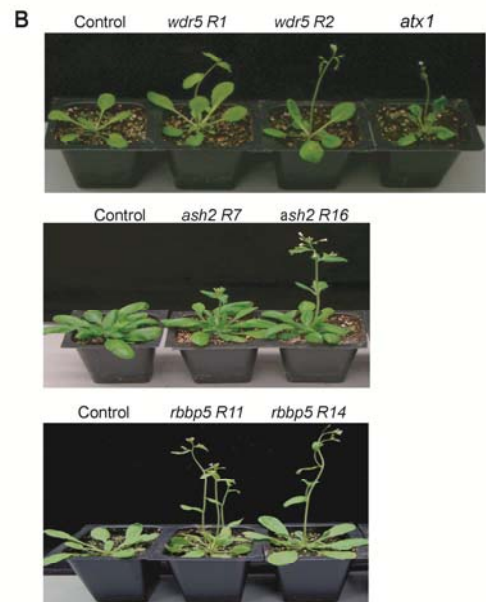

Supplement: Figure S1 — A) Relative mRNA levels in the two transgenic AtWDR5-RNAi (wR1 and wR2), two transgenic AtASH2-RNAi (a2R7 and a2R16), and two transgenic AtRbBP5-RNAi (rbR11, rbR14) lines used in this study. Transgenic plants transformed with the empty vector (Con) were used as a control; B) Flowering phenotypes in the RNAi-lines, in atx1, and control backgrounds. (PDF) [file pgen.1003111.s001.pdf]

Ding et al. Figure S2

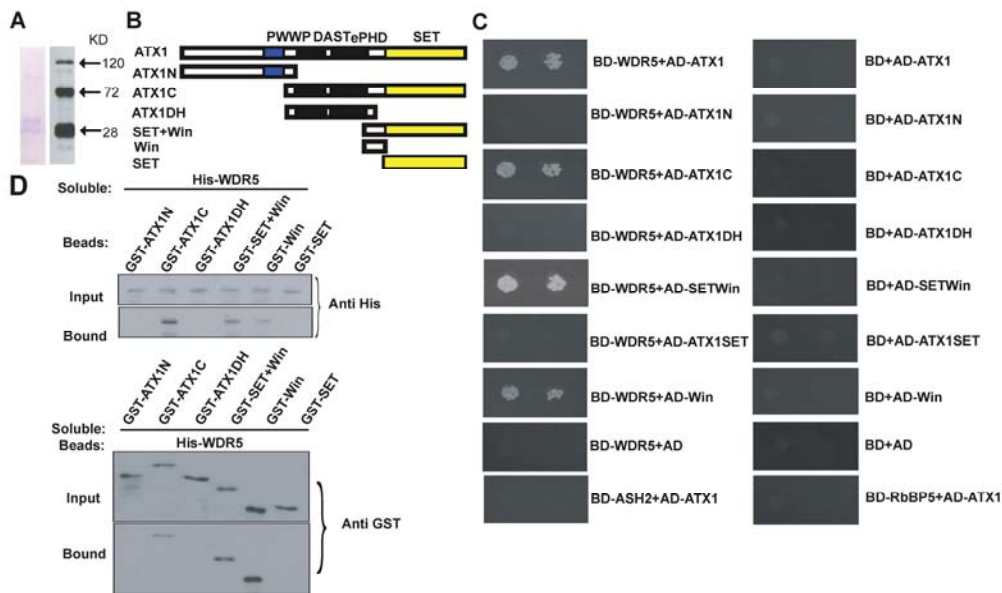

Supplement: Figure S2 — Interactions between ATX1, AtWDR5a, AtASH2, and AtRbPB5; Identification of the ATX1-Win AtWDR5-binding domain. A) Proteins from total cellular extracts retained by TAP-tagged-AtWDR5a in a pull-down experiment. Gel stained by Coomassie blue (left) and western blot assay with antiATX1 antibody (right). Arrows point to ATX1 (120 kD) and degradation products. Identities were established by MS (Figure S3); B) Schematic representation of ATX1 protein domains tested for their ability to bind AtWDR5a in a Y2-H system. Fragments corresponding to the ATX1 structural domains (PWWP, DAST, ePHD, Win, and SET) are indicated. Win (50 aa) represents a construct containing 50 amino acids upstream of the SET domain corresponding to the MLL1-Win peptide. C) Positive or negative interactions between ATX1 deletion fragments with AtWDR5a are indicated with (+) or (−), respectively. The experimental Y-2H data are shown on the right. The ATX1 proteins (shown in B) were fused to the activation domain (AD) and tested for their ability to bind to AtWDR5, AtASH2, or AtRbBP5, which were fused to the DNA binding domain (BD). Y-2H controls were performed with an AD only construct in combination with a BD containing construct (WDR5, ASH2, RbBP5, or BD alone) and the resulting growth or lack of growth of the yeast colonies is shown; D) In vitro pull-down assays of the domains tested in the Y2-H assays. Immobilized GST-tagged ATX1, or various ATX1-deletion fragments were tested for binding to soluble His-tagged AtWDR5 (top panel); Immobilized His-AtWDR5 was tested for binding to soluble GST-tagged ATX1 fragments (lower panel). Input and bound proteins were detected by anti-His tag or anti-GST antibody. (PDF) [file pgen.1003111.s002.pdf]

Ding et al. Figure S4

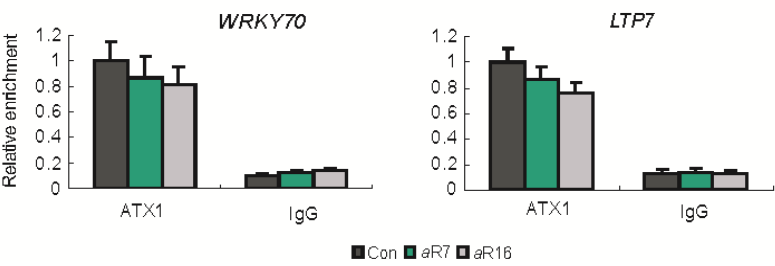

Supplement: Figure S4 — The amount of ATX1 at the promoter regions of three genes in AtASH2-deficient lines was determined by ChIP-PCR using antiATX1 antibody. The primers were located within the promoter regions (Region 1 in Figure 2A, main text). (PDF) [file pgen.1003111.s004.pdf]

Ding et al. Supplemental Figure 5

Figure S5

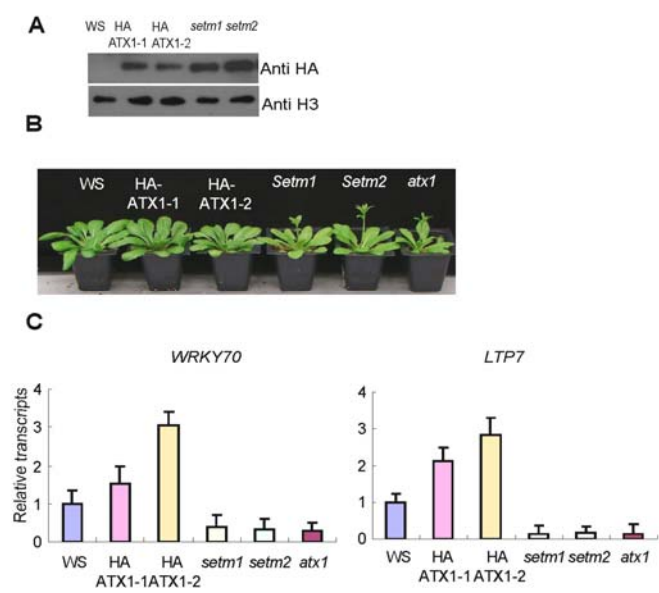

Supplement: Figure S5 — Transgenic atx1 mutant plants expressing the synthetic HA-tagged ATX1-wtSET domain protein or the HA-tagged ATX1 with Tyr/Ala substitutions in the SET domain (set). A) Western blot assay with antiHA antibody illustrating the expression of HA-ATX1-wtSET in two atx1 transgenic lines (HA-ATX1 and HA-ATX2) and in two atx1 lines expressing the HA-tagged ATX1-set mutant proteins (setm1 and setm2) transformed with the respective constructs. The levels of histone H3 expression detected by antiH3 antibodies are shown as loading controls; B) Flowering time phenotypes of plants from the transformed lines shown in (A). Transgenic atx1 plants expressing the HA-ATX1-setm do not rescue the early flowering caused by the atx1 mutation, while transformants expressing the wt SET restored wild type flowering; C) expression of the WRKY70 and LTP7 genes in set1 and set2 mutant lines in the atx1 background and in the atx1::HA-ATX1-wtSet background. The higher transcript levels from the WRKY70 and LTP7 genes may result from higher ATX1 protein produced in the transgenic lines. (PDF) [file pgen.1003111.s005.pdf]
