# Supplementary material for: ATX1-Generated H3K4me3 Is Required for Efficient Elongation of Transcription, Not Initiation, at ATX1-Regulated Genes
Source: PLoS Genet. 2012 Dec 20;8(12):e1003111. doi: 10.1371/journal.pgen.1003111 (PMC3527332; doi:10.1371/journal.pgen.1003111)
Supplement: Figure S3 — Identification of AtWDR5 interacting protein bands by MS. A) Coverage map of the ∼120 kD band reacting with the antiATX1 antibody (see arrow in Figure S2A). From MASCOT database search: Match to: gi|12659210 Score: 2058. Trithorax-like protein 1 [Arabidopsis thaliana]; Matched peptides shown in bold red. Score cut-off 35; B) Coverage map of the ∼72 kD band reacting with the antiATX1 antibody (see Figure 1A in text, arrowhead). From MASCOT database search: Match to: gi|12659210 Score: 3258. Trithorax-like protein 1 [Arabidopsis thaliana]; Matched peptides shown in bold red. Score cut-off 35; C) Coverage map of the ∼28 kD band reacting with the ATX1 antibody (see Figure 1A in text, arrow). From MASCOT database search: Match to: gi|12659210 Score: 863-Trithorax-like protein 1 [Arabidopsis thaliana]; Matched peptides shown in bold red. Score cut-off 35. Highlighted in yellow is the Win-homologous sequence; D) Alignment of the conserved Win seq upstream of the SET domain in MLL1 (Query) and ATX1 (Sbjct). The consensus ART sequence involved in the interaction with WDR5 is highlighted. (PDF) [file pgen.1003111.s003.pdf]

Ding et al., Figure S3 Identification of AtWDR5 interacting protein bands by MS

S3A: ATX1-120 kD

1 MACFSNETQI EIDVHDLVEA PIRYDSIESI YSIPSSALCC VNAVGSHSLM
51 SKKVKAQKLP MIEQFEIEGS GVSASDDCCR SDDYKLRIQR PEIVRVYYRR
101 RKRPLRECLL DQAVAVKTES VELDEIDCFE EKKRRKIGNC ELVKSGMESI
151 GLRRCKENNA FSGNK**QNGSS RRK**GSSSKNQ DKATLASRSA KKWVRLSYDG
201 VDPTSFIGLQ CKVFWPLDAL WYEGSIVGYS AERKRYTVKY RDGCDEDIVF
251 DREMIKFLVS REEMELLHLK FCTSNVTVDG RDYDEMVVLA ATLDECQDFE
301 PGDIVWAKLA GHAMWPAVIV DESIIGERKG LNNK**VSGGGS LLVQFFGTHD**
351 **FARIK**VKQAI SFIKGLLSPS HLKCKQPRFE EGMQEAKMYL KAHRLPERMS
401 QLQKGADSVD SDMANSTEEG NSGGDLLNDG EVWLRPTEHV DFR**HIIGDLL**
451 **IINLGKVVTD SQFFK**DENHI WPEGYTAMRK **FTSLTDHSAS ALYK**MEVLRD
501 AETK**THPLFI VTADSGEQFK GPTPSACWNK** IYNRIK**KVQN SDSPNILGEE**
551 **LNGSGTDMFG LSNPEVIKLV QDLSK**SRPSS HVSMCKNSLG RHQNQPTGYR
601 PVRVDWKDLD KCNVCHMDEE YENNLFLQCD KCRMMVHAKC YGELEPCDGA
651 LWLCNLCRPG APDMPPRCCL CPVVGGAMKP TTDGRWAHLA CAIWIPETCL
701 SDVKKMEPID GVNKVSKDRW KLMCTICGVS YGACIQCSNN SCRVAYHPLC
751 ARAAGLCVEL ENDMSVEGEE ADQCIRMLSF CKRHRQTSTA CLGSEDRIKS
801 ATHKTSEYLP PPNPSGCART EPYNCFGRRG RKEPEALAAA SSKRLFVENQ
851 PYVIGGYSRL EFSTYKSIHG SKVSQMNTPS NILSMAEKYR YMRETYRKRL
901 AFGKSGIHGF GIFAKLPHRA GDMMIEYTGE LVRPSIADKR EQLIYNSMVG
951 AGTYMFRIDD ERVIDATRTG SIAHLINHSC VPNCYSR**VIT VNGDEHIIIF**
1001 **AK**RHIPKWEE LTYDYRFFSI GERLSCSCGF PGCRGVVNDT EAEEQHAKIC
1051 VPRCDLIDWT AE

S3B: ATX1-72 kD
(DAST-PHD-SET)

441 DFR**HIIGDLL**
451 **IINLGKVVTD SQFFK**DENHI WPEGYTAMR**K FTSLTDHSAS ALYKMEVLRD**
501 **AETKTHPLFI VTADSGEQFK GPTPSACWNK** IYNRIKK**VQN SDSPNILGEE**
551 **LNGSGTDMFG LSNPEVIKLV QDLSKSRPSS HVSMCK**NSLG R**HQNQPTGYR**
601 **PVR**VDWKDLD KCNVCHMDEE YENNLFLQCD KCRMMVHAKC YGELEPCDGA
651 LWLCNLCRPG APDMPPRCCL CPVVGGAMKP TTDGRWAHLA CAIWIPETCL
701 SDVKKMEPID GVNKVSKDRW KLMCTICGVS YGACIQCSNN SCRVAYHPLC
751 ARAAGLCVEL ENDMSVEGEE ADQCIRMLSF CKRHRQTSTA CLGSEDRIKS
801 ATHK**TSEYLP PPNPSGCAR**T EPYNCFGRRG R**KEPEALAAA SSKRLFVENQ**
851 **PYVIGGYSRL EFSTYK**SIHG SK**VSQMNTPS NILSMAEK**YR YMRETYRKRL
901 AFGK**SGIHGF GIFAK**LPHR**A GDMMIEYTGE LVRPSIADKR EQLIYNSMVG**
951 **AGTYMFRIDD ERVIDATRTG SIAHLINHSC VPNCYSRVIT VNGDEHIIIF**
1001 **AK**R**HIPKWEE LTYDYRFFSI GERLSCSCGF PGCRGVVNDT EAEEQHAK**IC
1051 VPR**CDLIDWT AE**

S3C: ATX1-28 kD
(Win+SET)

810 ATHK**TSEYLP PPNPSGCART EPYNCFGR**RG RKEPEALAAA SSKR**LFVENQ**
851 **PYVIGGYSR**L EFSTYKSIHG SK**VSQMNTPS NILSMAEK**YR YMRETYRKRL
901 AFGKSGIHGF GIFAKLPHRA GDMMIEYTGE LVRPSIADKR EQLIYNSMVG
951 AGTYMFRIDD ERVIDATRTG SIAHLINHSC VPNCYSRVIT VNGDEHIIIF

1001 AKRHIPK**WEE LTYDYRFFSI GERLSCSCGF PGCRG**VVNDT EAEEQHAKIC
1051 VPRCDLIDWT AE

S3D

```
Query     PPLNPHGSARAEVHLRKSAFDMFNFLASKHRQPPE
          PP NP G AR E          +N+L  + R+ PE
Sbjct     PPPNPSGCARTEP---------YNCFGRRGRKEPE
```
