# Supplementary material for: ATX1-Generated H3K4me3 Is Required for Efficient Elongation of Transcription, Not Initiation, at ATX1-Regulated Genes
Source: PLoS Genet. 2012 Dec 20;8(12):e1003111. doi: 10.1371/journal.pgen.1003111 (PMC3527332; doi:10.1371/journal.pgen.1003111)
Supplement: Table S1 — Primers used for the various cloning and analytical procedures. (PDF) [file pgen.1003111.s006.pdf]

SUPPLEMEMNTARY TABLE 1: Primers used for the various cloning and analytical procedures.

**Pimer**	**5'-3' Sequence**	**Purpose**
WRKY70 Forward	AGGAGATGGGTTCGAAGGTA	*WRKY70* RT-PCR
WRKY70 Reverse	TCGTTGAAGGCCATGACTTA	*WRKY70* RT-PCR
LTP7 Forward	ATCACAGCAAAGGCGGETCTGAGC	*LTP7* RT-PCR
LTP7 Reverse	TACGTGTTGCACTTGGTGTTGAACC	*LTP7*RT-PCR
UBQ10 Forward	AGGATGGCAGAACTCTTGCT	*UBQ10* T-PCR
UBQ10 Reverse	TCCCAGTCAACGTCTTAACG	*UBQ10* RT-PCR
18S Forward	TCCTAGTAAGCGCGAGTCATCA	*18S* Nuclear run on PCR
18S Reverse	CGAACACTTCACCGGATCAT	*18S* Nuclear run on PCR
WRKY70 Forward	AGGAGATGGGTTCGAAGGTA	*WRKY70* Nuclear run on PCR
WRKY70 Reverse	TCGTTGAAGGCCATGACTTA	*WRKY70* Nuclear run on PCR
LTP7 Forward	ATCACAGCAAAGGCGGETCTGAGC	*LTP7* Nuclear run on PCR
*LTP7*Reverse	TACGTGTTGCACTTGGTGTTGAACC	*LTP7* Nuclear run on PCR
WRKY70 region 1 FD	AGATTTGTAATGATGGGCACTTT	*WRKY70* ChIP -PCR
WRKY70 region 1 RE	TCCATCTCATTTCCTCACACA	*WRKY70* ChIP -PCR
WRKY70 region 2 FD	AGGAGATGGGTTCGAAGGTA	*WRKY70* ChIP -PCR
WRKY70 region 2 RE	TCGTTGAAGGCCATGACTTA	*WRKY70* ChIP -PCR
WRKY70 region 3 FD	CTTCAAACTTGCCGTCGTTA	*WRKY70* ChIP -PCR
WRKY70 region 3RE	ATCTCCTCCTCCTCATCCCT	*WRKY70* ChIP -PCR
WRKY70 region 4 FD	TTTGTCCATATTTCCTCCAAGA	*WRKY70* ChIP -PCR
WRKY70 region 4 RE	AGCTTTGGTTGCAAGAATCA	*WRKY70* ChIP -PCR
WRKY70 region 5 FD	CACAAGTCTTGCTCTTGGGA	*WRKY70* ChIP -PCR
WRKY70 region 5 RE	GCAACAAAGCAAGTCCAGAA	*WRKY70* ChIP -PCR
WRKY70 region 6 FD	TCGCTAAACTCGAAATCGC	*WRKY70* ChIP -PCR
WRKY70 region 6 RE	CCAGTTACGTCAATGGGAAA	*WRKY70* ChIP -PCR
LTP7 region 1 FD	TGACCATTGTCTGTTGTCCGT	*LTP7* ChIP -PCR

LTP7 region 1 RE	TGCGTATGAAATTACGGTGAAACTC	*LTP7* ChIP -PCR
LTP7 region 2 FD	ATCACAGCAAAGGCGGCTCTGAGCT	*LTP7* ChIP -PCR
LTP7 region 2 RE	TACGTGTTGCACTTGGTGTTGAACC	*LTP7* ChIP -PCR
LTP7 region 3 FD	CAAGCATGTCGTTGCATTAAA	*LTP7* ChIP -PCR
LTP7 region 3 RE	GTTGCACTTGGTGTTGAACC	*LTP7* ChIP -PCR
Actin7 FD	CGTTTCGCTTTCCTTAGTGTTAGCT	*Actin7* ChIP -PCR
Actin7 RE	AGCGAACGGATCTAGAGACTCACCTTG	*Actin7* ChIP -PCR
WDR5 Forward	AGagatctCTATGGCAGAGGAAATTCCAGCAACA	*pGBDT7-WDR5*
WDR5 Reverse	GCagatctTTATTCTTTCTTCTGTGTCCAAATCCT	*pGBDT7-WDR5*
ATX1 Forward	ATAcccgggGATGGCGTGTTTTTCTAACGAAACCCA	*pGADT7-ATX1*
ATX1 Forward	CGAgtcgacTTATTCTGCGGTCCAGTCTATTAGA	*pGADT7-ATX1*
ATX1N Forward	GCTcatatgGCGTGTTTTTCTAACGAAACCCAG	*pGADT7-ATX1N*
ATX1N Reverse	GCGgaattcACTATTAGCCATATCGGAATCAACAG	*pGADT7-ATX1N*
ATX1C Forward	GTGgccatgGATTCCGATATGGCTAATAGTACAGA	*pGADT7-ATX1C*
ATX1C Reverse	GTGgccatgGTTCTGCGGTCCAGTCTATTAGATCACA	*pGADT7-ATX1C*
ATX1DH Forward	GTGcatatgGATTCCGATATGGCTAATAGTACAGA	*pGADT7-ATX1DH*
ATX1DH Reverse	AAAgcggccgcAGTTGATGTTTGTCGATG	*pGADT7-ATX1DH*
ATX1SET+Win Forward	CAGgaattcGAGAATGACATGTCAGTGGAGGGT	*pGADT7-ATX1SET+Win*
ATX1SET+Win Reverse	GTGGCCATGGTTCTGCGGTCCAGTCTATTAGATCACA	*pGADT7-ATX1SET+Win*
Win Forward	CAGgaattcGAGAATGACATGTCAGTGGAGGGT	*pGADT7- Win*
Win Reverse	GAGCACAGCCAGATGGATTA	*pGADT7- Win*
ATX1SET Forward	GCTcatatgAATACTCCAAGCAACATTCTTTC	*pGADT7-ATX1SET*
ATX1SET Reverse	TTCTGCGGTCCAGTCTATTAGATCACAACG	*pGADT7-ATX1SET*
RbBp5 Forward	ATGggatccGTATGAACGCTCCGATCATAGATCCATTG	*pGBDT7-RbBp5*
RbBp5 Reverse	GACggatccATAATATGCATCATCATCATCATCACCA	*pGBDT7-RbBp5*
Ash2 Forward	GCTgaattcATGGAGTCTCTTCAATCAAATTCCA	*pGBDT7-Ash2*
Ash2 Reverse	GCTgaattcACTCTTCATATCCTCAGAACCATTAG	*pGBDT7-Ash2*
WDR5 Forward	TAGagatctATGGCAGAGGAAATTCCAGCAACA	*pET28a-WDR5*
WDR5 Reverse	TGCagatctTTATTCTTTCTTCTGTGTCCAAATCCT	*pET28a-WDR5*
ATX1N Forward	GCTgaattcATGGCGTGTTTTTCTAACGAAACCCAG	*GST-ATX1N*
ATX1N Reverse	GCGgaattcACTATTAGCCATATCGGAATCAACAG	*GST-ATX1N*
ATX1C Forward	TGATTCCGATATGGCTAATAGTACAGA	*GST-ATX1C*
ATX1C Reverse	GTGGCCATGGTTCTGCGGTCCAGTCTATTAGATCACA	*GST-ATX1C*
ATX1DH Forward	TGATTCCGATATGGCTAATAGTACAGA	*GST-ATX1DH*
ATX1DH Reverse	AAAgcggccgcAGTTGATGTTTGTCGATG	*GST-ATX1DH*
ATX1SET+Win Forward	CAGgaattcGAGAATGACATGTCAGTGGAGGGT	*GST-ATX1SET+Win*
ATX1SET+Win Reverse	GTGGCCATGGTTCTGCGGTCCAGTCTATTAGATCACA	*GST-ATX1SET+Win*
Win Forward	CAGgaattcGAGAATGACATGTCAGTGGAGGGT	*GST- Win*
Win Reverse	GAGCACAGCCAGATGGATTA	*GST- Win*

ATX1 SET Forward	CACggatccAAGGTGTCACAGATGAA	*GST-ATX1 SET*
ATX1 SET Reverse	GTGGCCATGGTTCTGCGGTCCAGTCTATTAGATC ACA	*GST-ATX1 SET*
WDR Forward	GAagatctacgctgGTACGCTTCTCTTAAAAACCCAGC	*WDR5-RNAi*
WDR Reverse	CGggatccgcgcgcCTGCATTAAGTCATCACAAGTAGA	*WDR5-RNAi*
Ash2 Forward	CACCGAGGAGTTGTTGAAGGAGCTTGGT	*Ash2-RNAi*
Ash2 Reverse	TCACCTTCCTTGTACCCTTCCTCA	*Ash2-RNAi*
RbBp5 Forward	CACCTTGCTTTGGTTTCTGCGTCTGGTG	*RbBp5-RNAi*
RbBp5 Reverse	CCACTGAAACAAGGCGCTTTCCAA	*RbBp5-RNAi*
WDR Forward	CACCATGGCAGAGGAAATTCCAGCAACA	*TAP-WDR5*
WDR Reverse	TATTCTTTCTTCTGTGTCCAAATCCT	*TAP-WDR5*
