# Supplementary material for: ATX1-Generated H3K4me3 Is Required for Efficient Elongation of Transcription, Not Initiation, at ATX1-Regulated Genes
Source: PLoS Genet. 2012 Dec 20;8(12):e1003111. doi: 10.1371/journal.pgen.1003111 (PMC3527332; doi:10.1371/journal.pgen.1003111)
Supplement: Text S1 — Interaction between ATX1 and AtWDR5a; Identification of the ATX1-Win AtWDR5-binding domain. Interactions between ATX1 and AtASH2 or AtRbPB5. (PDF) [file pgen.1003111.s007.pdf]

SUPPORTING INFORMATION

Interaction between ATX1 and AtWDR5a; Identification of the ATX1-Win AtWDR5-binding domain

Whether AtWDR5 could bind ATX1 preferentially from the multitude of proteins present in total cellular extracts was determined first. A TAP-tagged AtWDR5 fusion protein was used as bait in the *in vitro* affinity-binding assay. The presence of ATX1 in the TAP-AtWDR5-bound protein fraction was detected by Western blot analysis with antiATX1-specific antibodies (Figure S2A) and the identities of the multiple-size fragments recognized by the antibody were confirmed by mass spectroscopy (Figure S3A-C). Detailed inspection revealed that all ATX1-related fragments retained on the AtWDR5 column contained the ATX1-SET domain plus upstream regions of a varying length. The smallest ATX1 degradation fragment bound by AtWDR5 contained an upstream region similar to the Win (WDR5-interacting) peptide of MLL1 [1-3] (Figure S3D).

To confirm this peptide was responsible for the interaction, a panel of ATX1 deletions was tested for their ability to bind AtWDR5 in the Y2-H binding system. The results showed that all ATX1 protein fragments able to interact with AtWDR5 contained the ATX1-Win peptide and that the ATX1-Win peptide alone could bind to AtWDR5 (Figure S2B, C), confirming this is the primary ATX1- AtWDR5 interaction region. The interactions were further confirmed by *in vitro* pull-down assays testing AtWDR5 and ATX1 derivatives as both baits and as substrates (Figure S2D).

Interactions between ATX1 and AtASH2 or AtRbPB5

The ability of ATX1 to interact with the other two complex subunits was tested in the Y2-H binding system under the same conditions used for the AtWDR5 assays. Lack of detectable growth suggested ATX1 did not bind directly to either AtASH2 or AtRbBP5 in the Y2-H binding system (Figure 2C). The results suggested that ATX1 integrates into the AtCOMPASS exclusively through the Win-mediated binding to AtWDR5.

Collectively, the results determined that AtWDR5 binds ATX1 within the cellular context. Interaction is via the ATX1-Win domain, a functional counterpart of the Win domain in MLL1 [1-3]. The significance of this result is that similarity of the interaction of the catalytic subunit with the rest of the complex implies functional similarity between the animal and plant complexes.

REFERENCES

1. Patel A, Dharmarajan V, Cosgrove MS (2008) Structure of WDR5 bound to mixed lineage leukemia protein-1 peptide. J Biol Chem 283: 32158-3261.
2. Song JJ, Kingston RE (2008) WDR5 interacts with mixed lineage leukemia (MLL) protein via the histone H3-binding pocket. J Biol Chem 283: 35258-35264.
3. Karatas H, Townsend EC, Bernard D, Dou Y, Wang S (2010) Analysis of the binding of mixed lineage leukemia 1 (MLL1) and histone 3 peptides to WD repeat domain 5 (WDR5) for the design of inhibitors of the MLL1-WDR5 interaction. J Med Chem 53: 5179-5185.
